# Understanding the manifestation of diabetes in sub Saharan Africa to inform therapeutic approaches and preventive strategies: a narrative review

**DOI:** 10.1186/s40842-019-0077-8

**Published:** 2019-02-14

**Authors:** Davis Kibirige, William Lumu, Angus G. Jones, Liam Smeeth, Andrew T. Hattersley, Moffat J. Nyirenda

**Affiliations:** 1 Non-Communicable Diseases Theme, Medical Research Council/Uganda Virus Research Institute and London School of Hygiene and Tropical Medicine Uganda Research Unit, Plot 51-59, Nakiwogo Road, P.O. BOX 49 Entebbe, Uganda; 2 Department of Medicine, Uganda Martyrs hospital Lubaga, Kampala, Uganda; 3 Department of Medicine, Mengo Hospital, Kampala, Uganda; 4 National Institute for Health Research, Exeter Clinical Research Facility, University of Exeter Medical School, Exeter, UK; 5 Department of Non-Communicable Diseases Epidemiology, London School of Hygiene and Tropical Medicine, London, UK

**Keywords:** Diabetes, Manifestation, Diabetes phenotype, Adult patients, Sub-Saharan Africa

## Abstract

**Background:**

Globally, the burden of diabetes mellitus has increased to epidemic proportions. Estimates from the International Diabetes Federation predict that the greatest future increase in the prevalence of diabetes mellitus will occur in Africa.

**Methods:**

This article reviews literature on the manifestation of diabetes in adult patients in sub-Saharan Africa highlighting the distinct phenotypes, plausible explanations for this unique manifestation and the clinical significance of comprehensively defining and understanding the African diabetes phenotype.

**Results:**

There are few studies on the manifestation or phenotype of diabetes in Africa. The limited data available suggests that, compared to the Western world, the majority of patients with diabetes in Africa are young and relatively lean in body size. In addition, hyperglycaemia in most cases is characterised by a significantly blunted acute first phase of insulin secretion in response to an oral or intravenous glucose load and pancreatic beta cell secretory dysfunction, rather than peripheral insulin resistance predominates. Genetic and environmental factors like chronic infections/inflammation, early life malnutrition and epigenetic modifications are thought to contribute to these distinct differences in manifestation.

**Conclusions:**

While published data is limited, there appears to be distinct phenotypes of diabetes in sub-Saharan Africa. Large and more detailed studies are needed especially among newly diagnosed patients to fully characterize diabetes in this region. This will further improve the understanding of manifestation of diabetes and guide the formulation of optimal therapeutic approaches and preventive strategies of the condition on the continent.

## Background

### Burden of diabetes: Globally and in Africa

Globally, the prevalence of diabetes mellitus (DM) has reached epidemic levels especially in low and middle income countries. According to the 2017 International Diabetes Federation (IDF) estimates, about 425 million adults have DM. This figure is projected to increase to 629 million adults by 2045, which is a 48% increase [1].

Africa is estimated to have 15.9 million adults living with DM which is a regional prevalence of 3.1%. The African continent has the greatest proportion of people with undiagnosed DM and global projections show that it will experience the greatest future increase in the burden of DM of about 156% by 2045 [1].

This growing burden of DM globally and in Africa has also been demonstrated by the pooled analysis of 751 population based studies performed in 146 countries from 1980 to 2014 by the Non-Communicable Diseases Risk Factor Collaboration (NCD-RisC) [2]. The global age-standardized diabetes prevalence increased from 4·3% (95% CI 2.4–7.0) in 1980 to 9·0% (95% CI 7.2–11.1) in 2014 in men and from 5% (95% CI 2.9–7.9) to 7.9% (95% CI 6.4–9.7) in women and worldwide, the number of adults with diabetes increased from 108 million in 1980 to 422 million in 2014. North Africa was one of the regions with the highest age standardized diabetes prevalence [2].

According to findings from the NCD-RisC Africa working group that analysed pooled data of 76 surveys (182,000 participants) from 32 countries performed between 1980 and 2014, the age standardized prevalence of DM increased from 3.4% (1.5–6.3) to 8.5% (6.5–10.8) in men, and from 4.1% (2.0–7.5) to 8.9% (6.9–11.2) in women [3]. The burden of DM was mostly higher in the Northern and Southern regions and a positive association was observed between mean body mass index (BMI) and diabetes prevalence in both sexes during that period [3].

The increasing dual burden of non-communicable diseases (NCD) like DM and communicable diseases such as HIV and tuberculosis puts a significant economic strain on the existing resource constrained health systems in sub-Saharan Africa (SSA). It also has huge economic implications for patients and their immediate families. It will therefore be crucial to fully understand how DM manifests in Africa to formulate and implement effective targeted preventive strategies and optimal management to reduce diabetes related morbidity and mortality.

## Methods

We searched PubMed, Google scholar, Scopus and African Journal Online databases for any published review articles, case reports and original research articles, regardless of year of publication that reported information about the manifestation of diabetes in adult patients in SSA emphasising mainly the reported distinct phenotypes. References of the identified publications were searched for more research articles to include in this narrative review.

The search terms used were: “manifestation of diabetes” OR “diabetes phenotypes” OR “presentation of diabetes” OR “characteristics of diabetes” OR “atypical diabetes” AND “Africa” OR “sub-Saharan Africa”.

We excluded research articles published in languages other than English and whose full texts were not accessible.

A total of 16 original articles, review articles and case reports containing information about the distinct diabetes phenotype in SSA were included in this narrative review [4–19].

## Results

### Manifestation of diabetes in sub Saharan Africa: Metabolic and immunologic characterization and atypical forms of diabetes

Both type 1 DM (T1DM) and type 2 DM (T2DM) are heterogeneous diseases that are characterized by a constellation of metabolic disorders and vary considerably in clinical presentation and disease progression [20].

A number of reports show that compared to high-income countries, the majority of adult patients diagnosed with DM in sub-Saharan Africa (SSA) are of median age of < 50 years (signifying early disease onset), lean body size (have low or normal BMI) and pancreatic beta cell secretory dysfunction characterised by a significantly blunted acute first phase of insulin secretion in response to an intravenous or oral glucose load predominates rather than peripheral insulin resistance [4–12].

This is in contrast with evidence reported from the Western world where DM appears to develop later in life (> 50 years), is common among overweight or morbidly obese individuals and increased IR, hyperinsulinaemia with progressive pancreatic beta cell secretory dysfunction occurring later in the course of the disease, as the hallmarks of DM [21, 22].

The observed predominant pancreatic beta cell secretory dysfunction might explain why DM in Africa is commonly associated with low BMI, keto-acidosis, rapid decline in fasting plasma C-peptide concentrations and early onset of secondary oral agent failure following diagnosis. The reasons for the above discussed differences in manifestation or diabetes phenotype are unclear, but may reflect genetic diversity and unique environmental factors in Africa.

Currently, there is a paradigm shift in the classification of DM from the definition based on need of insulin therapy to achieve euglycaemia and age of disease onset or diagnosis to a definition based on an in-depth definition of the underlying aetiopathogenetic mechanisms of hyperglycaemia [23]. This underscores the need to comprehensively describe and understand the phenotype of diabetes in SSA. This would help in guiding optimal and individualised management of patients with DM in clinical practice.

### Clinical and biochemical characterisation of diabetes in adult diabetic populations in sub-Saharan Africa

There have been very few published studies that have thoroughly investigated the clinical, metabolic and immunologic profile of African patients with DM. Despite this limitation, the available studies performed to demonstrate the distinct diabetes phenotype in SSA offer useful insights into the clinical and biochemical profile particularly with regard to the frequency of insulin resistance, beta cell secretory dysfunction and presence of pancreatic auto-immunity.

In one cross sectional study of 105 adult patients with DM conducted in the Tigray region of Northern Ethiopia (a semi-arid region), the mean age and median BMI was 41 ± 16 years and 20.6 (18.5–23.9) kg/m^2^ respectively [5], highlighting a young age at diagnosis and lean body size. Insulin deficiency expressed as C-peptide negative status on examination was reported in 43% of the patients with 28 and 3.8% of patients positive for glutamic acid decarboxylase antibodies (GADA) and islet antigen 2 antibody (IA-2A) tests respectively. About 38 (36%) patients had immunological and C-peptide characteristics that were not consistent with the classical T1DM and T2DM phenotypes, despite having clinical features similar to patients with T1DM (similar median age at diagnosis, glycated haemoglobin level and BMI). GADA positivity and C-peptide negativity in this sub-group was confirmed in 29 and 71% respectively [5]. This underscores the presence of diabetes phenotypes in SSA that may not fit the conventional classification of DM.

In another case control study that assessed the degree of basal insulin resistance (IR) and insulin secretion (IS) using the homeostatic model assessment (HOMA) among 146 patients with T2DM and 33 healthy controls performed in an urban hospital in Nigeria, IR and reduced IS prevalence among the T2DM patients was 95.5 and 74.7% respectively [7], demonstrating a high dual burden of IR and pancreatic beta cell secretory dysfunction. However, approximately 85% of the patients in this study were obese, which could explain the higher prevalence of IR. The independent predictors of IR in this study were age at diagnosis, waist circumference and duration of DM and those for reduced IS, duration of DM and waist circumference [7]. While the duration of DM in these patients was not reported, the prevalence of beta cell secretory dysfunction reported could probably have arisen as a result of beta cell exhaustion occurring as the disease progresses.

Another similar small study conducted in Nigeria comparing 40 patients with T2DM to 36 healthy controls reported an IR (defined as HOMA1-IR values > 1) prevalence of 87.5% in patients and 27.8% in the controls [8]. When a HOMA1-IR score ≥ 2 was used, IR was prevalent in 40% of the patients and 19.4% of the controls.

Two studies reported from Ghana reported severe pancreatic beta cell secretory dysfunction among a small adult population with DM [9, 10]. Amoah A et al. in their study that compared 15 healthy controls without family history of DM (group 1) with 11 healthy controls with first degree family history of DM (group 2) and 10 patients with T2DM (group 3) found that group 3 had severely blunted acute phases of insulin secretion following an intravenous glucose load as measured by the absolute and incremental area under the curve [10]. The mean acute first phase insulin secreted in the 1st, 2nd and 3rd group was 122 ± 75, 320 ± 11.7 and 7.8 ± 5.7 mU/l x minutes respectively with insulin sensitivity index lowest in the diabetic group [10].

### Plausible explanations for recognised distinct manifestation of diabetes in SSA

**Fig. 1:**
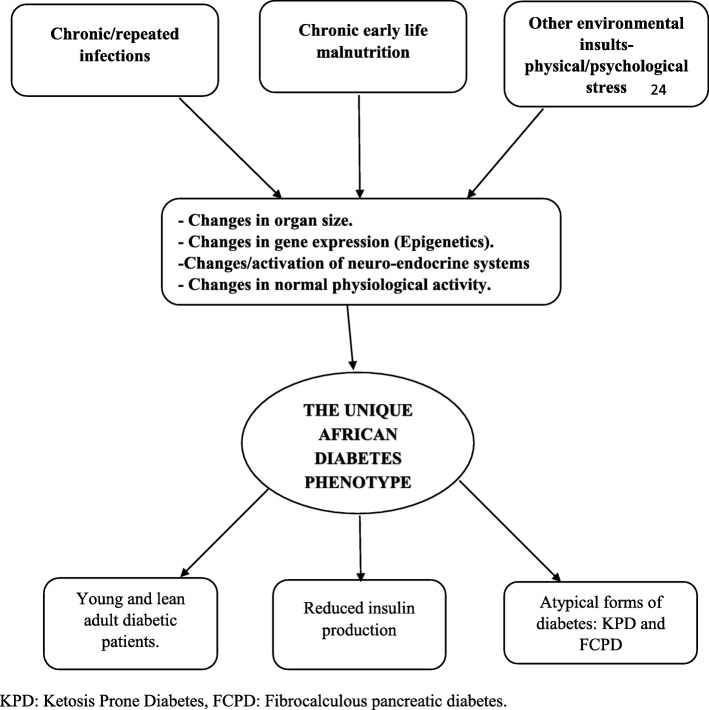
Plausible explanations for the distinct diabetes phenotype in sub Saharan Africa. KPD: Ketosis Prone Diabetes, FCPD: Fibrocalculous pancreatic diabetes

The African continent harbors the highest genetic heterogeneity. It is also unique because it continues to have a high burden of infectious diseases and other challenges like famine, civil strife and malnutrition. These exposures might modulate the pathogenesis and clinical course of NCD like DM, as summarized in Fig. 1.

There are clearly defined potential causes of the well documented distinct diabetes phenotype in SSA, while the others are less well investigated.

### Chronic infections and chronic pro-inflammatory state

Chronic infections notably HIV and tuberculosis (TB) that are highly endemic in SSA provoke a state of chronic inflammation which may play an important role in the pathogenesis of DM and other NCD, with proposed mechanisms including increased oxidative stress, vascular endothelial dysfunction and DNA damage [24]. This inflammatory cascade can lead to premature aging of key homeostatic organs like the pancreas and progressive decline of physiological functions. This eventually results into early onset of cardio-metabolic disorders like DM.

The pro-inflammatory state associated with infections like TB is also linked to increased release of counter regulatory stress hormones like cortisol and epinephrine which cause reactionary hyperglycaemia due to their antagonistic effects to insulin [25, 26]. The consequent reactionary hyperglycaemia can persist after TB treatment. The immune activation associated with HIV infection is also associated with a state of peripheral insulin resistance and reduced insulin secretion, hence increasing the risk of DM and metabolic syndrome at an early age [27].

### Infection induced hypersensitivity reaction/autoimmunity

Exaggerated immune responses (hypersensitivity reaction) and molecular mimicry as seen with some chronic infections have been implicated in pathogenesis of autoimmune conditions with subsequent organ dysfunction occurring especially in genetically predisposed individuals [28–30]. The underlying mechanisms explaining the hypersensitivity and autoimmunity induced by chronic infections include presence of antigenic mimicry, neo-antigen formation and immune dysregulation of the host [28].

Autoimmune destruction of pancreatic beta cells induced by enteroviruses like coxsackie B virus, rubella virus, rotavirus, flavivirus, herpes virus, rhinovirus and retrovirus have long been implicated as infectious aetiologies of T1DM [29–33].

### Direct organ damage by the pathogen

Direct pancreatic beta cell damage with subsequent endocrine dysfunction has also been highlighted as a potential mechanism of how infections can cause DM, impaired glucose tolerance at an early age and pancreatic beta cell secretory dysfunction. Tuberculous destruction of the beta and alpha cells of the pancreas, for example is often associated with brittle DM which is frequently characterised by labile blood glucose levels [25]. A close association has also been described between human herpes virus type 8, a virus endemic in the tropics responsible for causing Kaposi sarcoma and an atypical form of DM, ketosis prone diabetes (KPD) [13]. It is thought that it causes endoplasmic reticulum stress and direct destruction of the pancreatic beta cells which results in reduced insulin secretion [13].

### Effect of treatment of the infections

The HIV epidemic in SSA, coupled with increased access to antiretroviral therapy (ART) and longer life expectancy of HIV infected patients have been associated with development of multiple metabolic derangements [34] and could also partly explain the distinct phenotypes of diabetes in SSA.

Anti-retroviral therapy notably efavirenz and protease inhibitors have been shown to increase the risk of dysglycaemia [35–37]. Unequivocal evidence shows that these classes of ART cause mitochondrial dysfunction, significant changes in body fat distribution (increased central adiposity and peripheral lipoatrophy) and reduce cellular uptake of glucose by impairing the activity of the glucose transporter-4 [27, 36–39].

Rifampicin, one of the potent anti TB drugs is also associated with a transient hyperglycaemia that may persist during and after treatment because of its effect of augmenting intestinal glucose absorption [25].

### Chronic prenatal and postnatal malnutrition (thrifty phenotype hypothesis)

Chronic prenatal and postnatal malnutrition frequently seen in most parts of SSA especially in the rural areas is a recognised precursor for DM and other NCD and could also explain the distinct diabetes phenotypes seen in SSA. The link between perinatal malnutrition during periods of famine and increased odds of developing DM later in life has been demonstrated in studies reported from Ukraine [40] and Ethiopia [41].

The thrifty phenotype hypothesis which was described several decades ago by Nicholas Hales and David Barker expounds on the link between chronic perinatal malnutrition which manifests as low birth weight and failure to thrive with subsequent development of NCD like T2DM in adulthood [42]. One hypothesis to explain the future development of T2DM in patients with a history of early life malnutrition is possibly impaired development, innervation and function of pancreatic beta cell mass and islet of Langerhans [42, 43]. Fetal malnutrition exacerbates the risk of IR and obesity later in life in cases of reversed (nutrient rich) environment and positive calorie balance due to increased food intake and decreased energy expenditure [44].

This increased susceptibility to NCD like DM due to early life exposure and changes in adult lifestyles due to globalisation is what is essentially seen in SSA.

Vitamin D deficiency which is caused by malnutrition and other factors like chronic infections like TB, HIV and dark skin pigmentation may also explain the increased the odds of developing DM and the unique manifestation in SSA [45]. Some of the integral roles of vitamin D are increasing pancreatic beta cell production of insulin by increasing intra-cellular calcium concentrations, activation of intra cellular endopeptidases that cleave pro-insulin to insulin and by preventing inflammatory damage of pancreatic beta cells [46].

### Epigenetic modifications

Findings from both animal studies and recent large scale human epigenome wide associated studies show epigenetics as the common link between genome and environmental factors like chronic malnutrition and the development of DM [47–49]. These could explain the uniqueness in diabetes phenotypes in SSA.

According to the Developmental Origins of Health and Disease fetal origins of adult disease hypothesis, in-utero fetal programming induced by exposures to malnutrition, stress and fetal infections like malaria and toxoplasmosis, rubella, CMV, herpes simplex and syphilis (TORCHES) results in short and long term adaptations which are partly mediated by epigenetic changes. These adaptations are essentially to ensure fetal survival [50].

Epigenetic changes ranging from DNA methylation, histone modification and noncoding RNAs occur during development, are transmitted from cell to cell (mitotic inheritance) or generation to generation (transgenerational epigenetic inheritance) and cause alteration of gene expression, cellular growth, composition and physiology [51].

These epigenetic changes result in simple organ failure (reduction in cellular size and number), alteration in endocrine systems (upregulation of the hypothalamic-pituitary-adrenal axis and changes in secretion and sensitivity to insulin and insulin-like growth factor-1) and changes in expression and regulation of DNA [52]. Epigenetic modifications that result in reduced pancreatic beta cell mass and function coupled with changes in cellular insulin signaling, reduced muscle mass and increased adiposity lead to an increased likelihood of development of DM and could partly explain the unique diabetes phenotype seen in SSA.

### Changes or activation of the neuro-endocrine systems

Environmental insults such as maternal infection, stress and malnutrition have been shown to activate the hypothalamus pituitary adrenal axis with resultant increase in the expression of the glucocorticoid receptors, dampening of the hypothalamic negative feedback mechanism and increased production of stress hormones (glucocorticoids) by the adrenal glands [53, 54].

Studies in rat models have demonstrated that high fetal glucocorticoid levels due to stressful states or environmental insults like malnutrition attenuate the expression and activity of the placental enzyme 11β-hydroxysteroid dehydrogenase type-2 that is key in modulating fetal exposure to glucocorticoids [55, 56]. The downregulation of this enzyme subsequently is associated with early onset of glucose intolerance, hypertension and other cardiovascular diseases in adulthood.

### Atypical forms of diabetes in Africa: Fibrocalculous pancreatic diabetes and ketosis prone diabetes

The distinctiveness in the diabetes phenotype in SSA as explained by the discussed factors above is further emphasized by the presence of these 2 unique atypical forms of the diabetes i.e. KPD and fibrocalculous pancreatic diabetes (FCPD) which have been described particularly among patients of African ancestry [12, 14–18]. Despite being exclusively described in African populations, we lack population based studies in the region investigating the prevalence of these atypical sub-types.

### Ketosis prone diabetes (KPD)

Patients with KPD present with acute severe hyperglycaemia and keto-acidosis but, in contrast to classic T1DM lack pancreatic islet beta cell auto antibodies or a genetic association with HLA [19]. The defects in pancreatic beta cell function and insulin sensitivity at presentation in this condition remarkably improve with insulin therapy, and many patients can discontinue insulin following treatment of the acute episode, with near normoglycaemic remission that may last from months to years [19]. This insulin free period in patients with KPD is similar to the well described “honeymoon period” seen in patients with T2DM [57] and T1DM [58], which is a drug free period with observed sustained optimal glycaemic control. This clinical observation is explained by the resumption of endogenous insulin production by the pancreatic beta cells after glucotoxicity is resolved following acute intensive insulin therapy [59].

### Fibrocalculous pancreatic diabetes (FCPD)

FCPD is one of the unique forms of malnutrition related DM that develops secondary to non-alcoholic chronic calcific pancreatitis and has been widely described in the tropical developing countries in SSA [14, 18]. It is diagnosed mainly among young patients presenting with abdominal pain, radiological confirmation of pancreatic calcification and features of pancreatic exocrine (steatorrhea) and endocrine (severe hyperglycaemia) insufficiency [60]. Patients with FCPD present with early disease onset (< 30 years), an associated male preponderance (70%), are underweight, of a low socio-economic status and have severe hyperglycaemia that requires insulin therapy in low doses to achieve euglycaemia [18, 60]. Diabetic keto-acidosis rarely develops following insulin withdrawal because of partial preservation of pancreatic beta cell function as evidenced by relatively normal C-peptide levels, low glucagon levels and decreased adipose tissue mass [60].

## Discussion

The current paradigm shift in the classification of DM underscores the need to understand the underlying pathophysiologic mechanisms of hyperglycaemia because of their potential therapeutic implications [23]. Evidence based management of DM recommends the use of combination therapy based on the defined underlying pathophysiological abnormalities or defects to optimize management [23, 61].

From an African perspective, an in-depth understanding of the underlying pathophysiologic defects and manifestation of DM offers important insights about the optimal drug combinations or prevention strategies for management and prevention of DM in this specific patient population. These insights can also offer a platform for future interventional studies to investigate which pharmacotherapy (monotherapy or in combination) would be optimal in managing hyperglycaemia, preservation of beta cell function or retarding progressive beta cell failure in an African adult population with DM, and may inform lifestyle advice and other strategies to prevent or screen for diabetes.

Most clinicians in SSA manage their adult patients with DM basing on guidelines developed by international diabetes associations like American Diabetes Association [23]. These international guidelines are developed based on evidence from phenotyping studies of Caucasian or mixed ancestry populations, and may therefore not be applicable and need to be cautiously extrapolated to other populations.

An example is the recommendation to use metformin as the first line therapy in the management of DM in adult patients [23]. Metformin is currently the first choice of therapy for T2DM in international guidelines and it is the most commonly used first line therapy in clinical practice in SSA. While there is clear evidence for metformin being the optimal therapy in obese western populations with T2DM, there is no evidence to show this is the optimal first line treatment in African populations who have a completely different phenotype.

## Conclusion

To address the paucity of clinical studies describing the diabetic phenotype of native African patients, more large studies comprehensively assessing the clinical and metabolic profile of adult patients especially among newly diagnosed patients with DM are warranted to further improve the understanding of the manifestation of DM in SSA. This will be key in informing the formulation of optimal therapeutic options and targeted preventive strategies for DM that are individualised for the African population.

## Abbreviations

BMI: Body mass index
DM: Diabetes mellitus
FCPD: Fibrocalculous pancreatic diabetes
GADA: Glutamic acid decarboxylase antibodies
GC: glucocorticoid
HOMA: Homeostatic model assessment
IA-2A: Islet antigen 2 antibody
IDF: International Diabetes Federation
IR: Insulin resistance
IS: Insulin secretion
KPD: Ketosis prone diabetes
NCD: Non-communicable diseases
NCD-RisC: Non-Communicable Diseases Risk Factor Collaboration
SSA: Sub-Saharan Africa
T1DM: Type 1 DM
T2DM: Type 2 DM
TB: Tuberculosis
